# The effects of incidental findings from whole-body MRI on the frequency of biopsies and detected malignancies or benign conditions in a general population cohort study

**DOI:** 10.1007/s10654-020-00679-4

**Published:** 2020-08-29

**Authors:** Adrian Richter, Elizabeth Sierocinski, Stephan Singer, Robin Bülow, Carolin Hackmann, Jean-François Chenot, Carsten Oliver Schmidt

**Affiliations:** 1 Department SHIP-KEF, Institute for Community Medicine, Greifswald University Medical Center, Walther Rathenau Str. 48, 17475 Greifswald, Germany; 2 Department of Family Medicine, Institute for Community Medicine, Fleischmannstr. 42, 17475 Greifswald, Germany; 3 Institute for Pathology, Greifswald University Medical Center, Friedrich-Loeffler-Str. 23e, 17487 Greifswald, Germany; 4 Institute of Pathology, University Hospital Tuebingen, Liebermeisterstrasse 8, 72076 Tuebingen, Germany; 5 Department of Diagnostic Radiology and Neuroradiology, Greifswald University Medical Center Greifswald, 17475 Greifswald, Germany; 6 Institute for Hygiene and Environmental Medicine, Charité – University Medicine Berlin, Hindenburgdamm 27, 12203 Berlin, Germany

**Keywords:** Magnetic resonance imaging, Incidental findings, Biopsies, Histological examinations, Record linkage

## Abstract

Magnetic resonance imaging (MRI) yields numerous tumor-related incidental findings (IFs) which may trigger diagnostics such as biopsies. To clarify these effects, we studied how whole-body MRI IF disclosure in a population-based cohort affected biopsy frequency and the detection of malignancies. Laboratory disclosures were also assessed. Data from 6753 participants in the Study of Health in Pomerania (SHIP) examined between 2008 and 2012 were utilized. All underwent laboratory examinations and 3371 (49.9%) a whole-body MRI. Electronic biopsy reports from 2002 to 2017 were linked to participants and assigned to outcome categories. Biopsy frequency 2 years pre- and post-SHIP was investigated using generalized estimating equations with a negative-binomial distribution. Overall 8208 IFs (laboratory findings outside reference limits: 6839; MRI: 1369) were disclosed to 4707 participants; 2271 biopsy reports belonged to 1200 participants (17.8%). Of these, 938 biopsies occurred pre-SHIP; 1333 post-SHIP (event rate/100 observation years = 6.9 [95% CI 6.5; 7.4]; 9.9 [9.3; 10.4]). Age, cancer history, recent hospitalization, female sex, and IF disclosure were associated with higher biopsy rates. Nonmalignant biopsy results increased more in participants with disclosures (post-/pre-SHIP rate ratio 1.39 [95% CI 1.22; 1.58]) than without (1.09 [95% CI 0.85; 1.38]). Malignant biopsy results were more frequent post-SHIP (rate ratio 1.74 [95% CI 1.27; 2.42]). Biopsies increased after participation in a population-based cohort study with MRI and laboratory IF disclosure. Most biopsies resulted in no findings and few malignancies were diagnosed, indicating potential overtesting and overdiagnosis. A more restrictive policy regarding IF disclosure from research findings is required.

**Electronic supplementary material:**

The online version of this article (10.1007/s10654-020-00679-4) contains supplementary material, which is available to authorized users.

## Introduction

The challenge of managing incidental findings (IFs) in clinical practice and research is growing with the increasing accessibility of powerful imaging modalities such as MRI [1–3]. IFs frequently occur in clinical as well as in research settings [1, 2, 4]. The use of whole-body MRI (wb-MRI) in a population-based cohort of 2500 participants resulted in 13,455 IFs, of which 1330 were potentially clinically relevant and disclosed to participants [1].

Between 50 and 80% of IFs from research MRI are suspicious for malignancy [1, 3]. Such findings may enable timely treatment of a disease, offering potential improvement or preservation of quality and length of life [5, 6]. On the other hand, overtesting and overdiagnosis may result [7], incurring additional costs to the health care system as well as psychosocial costs for patients who anxiously await results or are faced with findings of unknown relevance [8, 9].

Despite the frequency of these potentially significant findings, information regarding the clinical outcomes of IFs is limited [3]. There exists uncertainty as to which IFs warrant disclosure and further investigation. Management guidelines are lacking [3, 10, 11]. Research participants with disclosed IFs may present to their physicians and invasive diagnostic actions such as biopsies may result to rule out serious pathology. Histological examinations of biopsied tissue are the most effective but also the most invasive way to obtain diagnostic certainty. Individuals undergoing biopsy are exposed to potential discomfort, pain, and complications such as infection, bleeding, damage to nearby structures, or tumor seeding [12–16]. Other adverse consequences include potential costs, side effects, and complications of clinically unnecessary therapeutic interventions resulting from overdiagnosis [7, 17]. Such consequences are particularly undesirable in the context of observational research designs as they may bias longitudinal study data of health related outcomes.

To the best of our knowledge, there exists no longitudinal data on the effects of IFs on the frequency and outcomes of biopsies. The aim of this study was therefore twofold. First, we assessed whether the disclosure of incidental wb-MRI findings in a population-based cohort study was associated with an increase in biopsy frequency. The number of biopsies during the 2 years prior to examination in a large population-based cohort study was compared to the number 2 years after participation, adjusted for socio-demographic and clinical characteristics. Second, we analyzed whether the disclosure of IFs contributed to the detection of new malignancies via biopsy. We accounted for the concomitant effects of disclosed laboratory results because of their potential role in the decision to biopsy.

## Methods

### Data sources

This study uses data from the Study of Health in Pomerania (SHIP) and histology data from the Greifswald University Medical Center Department of Pathology. SHIP design and methods are described in detail in other publications [18]; a summary is provided below.

### SHIP cohorts

SHIP is a population-based project consisting of two independent cohorts, SHIP and SHIP-TREND. Participants were selected from northeastern Germany [18]. Out of 6265 eligible individuals of the first cohort, 4308 (2192 women) participated (response 68.8%) in the SHIP-0 baseline examination [19]. Baseline examinations were performed from 1997 to 2001. Follow-up examinations took place between 2002 and 2006 (SHIP-1, N = 3300) and between 2008 and 2012 (SHIP-2, N = 2333).

A second cohort, SHIP-Trend-0, was established in 2008. A stratified sample of 10,000 was drawn from the central population registry. Out of the net sample of 8826, after exclusion of deceased and relocated participants, 4420 (2275 women) participated (response 50.1%) in the baseline examination between 2008 and 2012.

All analyses are based on data from the SHIP-2 and SHIP-Trend-0 examinations, which were conducted in parallel and included among others extensive laboratory investigations, a personal interview about medical history, socio-demographics, and a whole-body MRI. The full scope of examinations is described elsewhere [18].

**Fig. 1:**
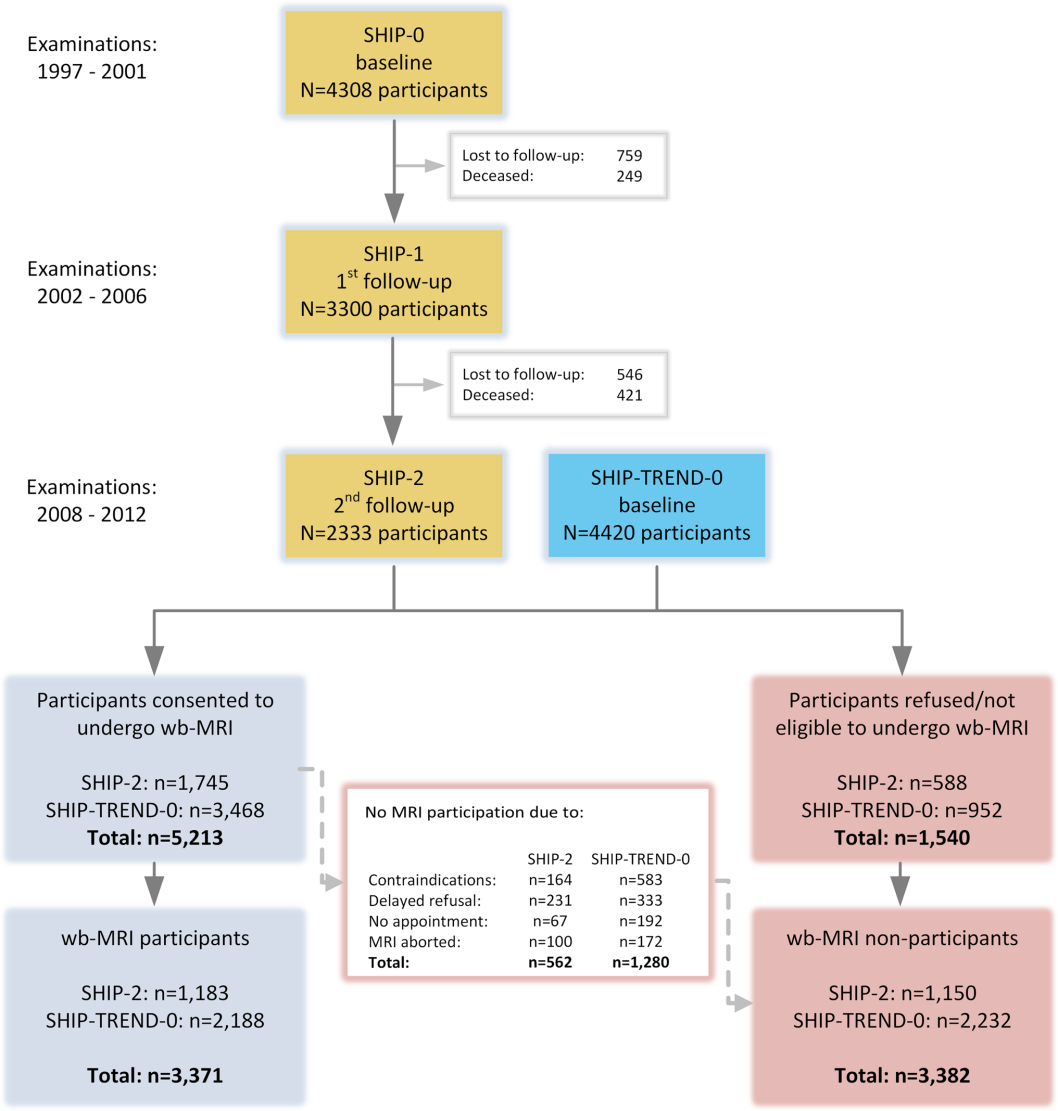
Study flow chart

All SHIP-2 and SHIP Trend participants were invited to take part in a whole-body MRI examination, with the exception of 527 (SHIP-2: n = 57, SHIP-TREND-0: n = 467) who received a brief examination in remote examination centres. For the latter no appointment for MRI was arranged. A total of 3371 individuals participated in the MRI examination and 3382 did not. A detailed overview is provided in the flow-chart (Fig. 1).

#### Whole-body MRI

A 1·5-Tesla system (Magnetom Avanto; Siemens Medical Solutions, Erlangen, Germany) was used for wb-MRI. The wb-MRI protocol was identical for all participants and included a plain whole-body MRI with detailed imaging of the head, neck, chest, abdomen, pelvis, and spine. Men had the option of contrast-enhanced cardiac MRI and MR angiography, and women had the option of cardiac MRI and contrast-enhanced MR mammography. The complete imaging protocols have been described previously [1, 20].

Abnormal findings and anatomical variants were documented in a standardized reading protocol. The radiologists reading the scans had no access to participants’ clinical information. Scan reading was performed using a digital picture archiving and communication system (IMPACS ES 5·2, AGFA Healthcare, Mortsel, Belgium). First-line reading was performed by two independent radiology residents. A third reader, a senior radiologist with 15 years of experience, resolved disagreements [1].

#### Laboratory examination

Venous blood and urine samples were taken from all study participants. Serum aliquots were stored at – 80 °C. The laboratory in charge for SHIP blood samples takes part in the official German external quality proficiency testing program. All assays are calibrated against the international reference preparations. A list of all covered biomarkers is provided elsewhere [18].

Parameters with the potential to trigger a biopsy were chosen on clinical grounds by JFC and ES: alanine transaminase (ALT), aspartate transaminase (AST), gamma-glutamyl transpeptidase (GGT), lipase, serum white blood cells (WBC), serum platelets (PLT), thyroid stimulating hormone (TSH), and urine erythrocyte count. Parameters exceeding reference limits were summarized in a variable indicating whether one or more laboratory results exceeded the limits.

#### Disclosure of incidental findings from imaging and laboratory

A standardized protocol, approved by the institutional review board, regulated the handling of wb-MRI incidental findings. Findings were classified into three categories by trained radiologists: Category I comprised medically non-significant findings in asymptomatic individuals (e.g., anatomical variants, old brain infarcts). Category II findings were abnormalities needing further non-urgent medical evaluation (e.g., tumors or nodules of unclear significance). Category III included urgent findings requiring immediate referral (e.g., acute brain infarcts, fractures, lobar pneumonia). Category II findings were disclosed to participants by means of a postal letter after approval by an interdisciplinary advisory board. This included a recommendation to seek further medical assistance. Category III findings were disclosed immediately to the participant after the examination. A more detailed description of the process and the frequencies of category II and III findings has been provided elsewhere [1, 20].

All participants received a paper copy of their laboratory results. Laboratory values crossing reference limits were highlighted [18].

### Linkage of histological data with SHIP data

Data from the pathology department and the SHIP study have no common key for linkage. Therefore we applied record linkage as discussed in Vatsalan et al. [21] based on: last name, first name, date of birth, and sex given the consent of participants. The linkage process included a normalization of personal data (to upper case letters, removal of special characters), the indexing of candidate pairs based on birth date, and the comparison. Candidate pairs were compared using generalized Levenshtein distances [22]. Respective R-Code was parallelized and blocked via birth date to reduce computational costs [23, 24]. In total, 3340 SHIP participants were linked based on a Levenshtein distance of zero. In 422 participants with a Levenshtein distance > 0, a manual revision for transposition, omission, or addition of single characters in their personal data identified additional 149 matches. Out of the 3489 participants with biopsy records, 1200 had at least one biopsy report in the analysis period (± 2 years from SHIP examination).

#### Classification of biopsy reports

From the database of the Greifswald University Medical Center Department of Pathology a total of 8576 histological reports from SHIP participants were available, dating from 2002 to 2019. The histological reports were unstructured and available in free-text format. They contained varying levels of detail regarding clinical history, macro- and microscopy, and differential/excluded/final diagnosis.

Three (0.1%) autopsies were excluded and 56 (1.8%) reports were not classifiable due to missing or incomplete data. Twenty-five reports (0.8%) included two different tissue types and were assigned to two outcome categories. In total, 3011 biopsy reports were included and classified. Of these, 2271 were dated within the analysis period, i.e., ± 2 years to the SHIP examination, and 740 represented earlier biopsy reports from patients with at least one report within the analysis period. Reports from the pre-analysis period were included when a report within the analysis period occurred. This ensured the correct classification of 1st and 2nd malignancies as well as follow-up biopsies.

The outcome or diagnosis detailed in each biopsy report was classified into mutually exclusive categories: pre-cancer; 1st to 5th malignancy; metastasis; benign tumor; follow-up of known malignant or suspicious process; no tumor or malignancy; updated pathological report; no diagnosis possible.

ES classified the entire set of biopsies. Double-readings were conducted for 2510 (83.4%) biopsies (CH: n = 1752, JFC: n = 758). Dissent was resolved by consensus readings and by consulting a pathologist (StS). Overall, dissent between readers was observed in 239 of 2510 double-classified reports (9.5%). In total, 58 corrections of initial classifications done by ES (2.3% of 2510 double readings) were revoked by the consensus decision. Therefore, we assume a misclassification rate of lower than 3% in the 501 single-readings.

### Statistical analyses

Baseline characteristics were stratified for the respective cohort and participation of MRI examination. Descriptive measures for location (mean, median) are shown with standard deviation (SD), minimum, and maximum. Categorical data was described using the number of events (N) and percentage. Missing values for all variables are provided.

Crude event rates (per 100 observation years) of biopsies were calculated using median unbiased estimates and exact mid-p 95% confidence intervals [25, 26]. The observation period covered 2 years before and after the SHIP examination. In addition, the frequency of biopsies (count data) was estimated using a generalized estimating equation model with a negative binomial distribution. Based on the QIC statistic we chose an independent working correlation [27]. Coefficients of the model were exponentiated for interpretation as incidence rate ratios [28]. Variable selection was based on clinical appraisal of relevance a priori. Age, sex, socio-demographic characteristics (education, relationship status), hospitalization within last 12 months, history of cancer, and the disclosure of incidental findings were included in the model. Due to the small amount of missing values (max. 1.9% for history of cancer, Table 1) no imputation techniques were applied and complete case analyses were conducted.

**Table 1:** Study population characteristics

Characteristics	SHIP-BASE (MRI)	SHIP-BASE (no MRI)	SHIP-TREND (MRI)	SHIP-TREND (no MRI)	All
N	1183	1150	2188	2232	6753
Age (years)					
Mean (SD)	55.7 (12.8)	59.0 (14.3)	51.2 (14.1)	52.7 (16.7)	53.8 (15.1)
Median [Min, Max]	56.0 [30.0, 90.0]	59.0 [31.0, 93.0]	52.0 [21.0, 82.0]	54.0 [20.0, 84.0]	54.0 [20.0, 93.0]
Sex					
Female	605 (51.1%)	630 (54.8%)	1113 (50.9%)	1162 (52.1%)	3510 (52.0%)
Male	578 (48.9%)	520 (45.2%)	1075 (49.1%)	1070 (47.9%)	3243 (48.0%)
Educational level					
Normal/high	856 (72.4%)	685 (59.6%)	1847 (84.4%)	1556 (69.7%)	4944 (73.2%)
Lower	327 (27.6%)	463 (40.3%)	337 (15.4%)	667 (29.9%)	1794 (26.6%)
Missing	0 (0%)	2 (0.2%)	4 (0.2%)	9 (0.4%)	15 (0.2%)
Years of education					
< 10	253 (21.4%)	390 (33.9%)	344 (15.7%)	685 (30.7%)	1672 (24.8%)
10	652 (55.1%)	562 (48.9%)	1178 (53.8%)	1090 (48.8%)	3482 (51.6%)
> 10	278 (23.5%)	193 (16.8%)	662 (30.3%)	448 (20.1%)	1581 (23.4%)
Missing	0 (0%)	5 (0.4%)	4 (0.2%)	9 (0.4%)	18 (0.3%)
Marital status					
Single	129 (10.9%)	114 (9.9%)	223 (10.2%)	262 (11.7%)	728 (10.8%)
In a relationship	965 (81.6%)	900 (78.3%)	1755 (80.2%)	1663 (74.5%)	5283 (78.2%)
Divorced	55 (4.6%)	76 (6.6%)	128 (5.9%)	155 (6.9%)	414 (6.1%)
Widowed	34 (2.9%)	57 (5.0%)	78 (3.6%)	143 (6.4%)	312 (4.6%)
Missing	0 (0%)	3 (0.3%)	4 (0.2%)	9 (0.4%)	16 (0.2%)
Employment status					
Unemployed	523 (44.2%)	649 (56.4%)	913 (41.7%)	1240 (55.6%)	3325 (49.2%)
Employed	658 (55.6%)	497 (43.2%)	1271 (58.1%)	980 (43.9%)	3406 (50.4%)
Missing	2 (0.2%)	4 (0.3%)	4 (0.2%)	12 (0.5%)	22 (0.3%)
Hospitalized in last 12 months					
No	1014 (85.7%)	919 (79.9%)	1905 (87.1%)	1864 (83.5%)	5702 (84.4%)
Yes	166 (14.0%)	227 (19.7%)	280 (12.8%)	360 (16.1%)	1033 (15.3%)
Missing	3 (0.3%)	4 (0.3%)	3 (0.1%)	8 (0.4%)	18 (0.3%)
Cancer history					
No	1092 (92.3%)	1071 (93.1%)	2047 (93.6%)	1957 (87.7%)	6167 (91.3%)
Yes	89 (7.5%)	78 (6.8%)	135 (6.2%)	153 (6.9%)	455 (6.7%)
Missing	2 (0.2%)	1 (0.1%)	6 (0.3%)	122 (5.5%)	131 (1.9%)
Histological data available					
No	979 (82.8%)	940 (81.7%)	1808 (82.6%)	1826 (81.8%)	5553 (82.2%)
Yes	204 (17.2%)	210 (18.3%)	380 (17.4%)	406 (18.2%)	1200 (17.8%)
Laboratory abnormalities					
No	492 (41.6%)	382 (33.2%)	832 (38.0%)	736 (33.0%)	2442 (36.2%)
Yes	691 (58.4%)	768 (66.8%)	1356 (62.0%)	1496 (67.0%)	4311 (63.8%)

The cumulative rates of biopsies were investigated using recurrent event analysis. Biopsy outcomes such as “no malignant finding” or “follow up of known malignant process” could occur multiple times for each participant. The 1st to 5th malignancies as well as metastases were summarized in one category of recurrent events of the same entity. The cumulative rate of recurrent events was represented graphically using the R-package reReg [29]. The R-package reReg allows for the computation of cumulative hazard rate using the Nelson-Aalen estimator [30] without adjusting the risk set in case of an event.

Record linkage and the analysis of recurrent events were conducted using the statistical software R [31]. For data pre-processing and the GEE models SAS version 9.4 (SAS Institute Inc., Cary, NC, USA) was used. We present all effects including confidence intervals [32, 33]. Confidence intervals and effect sizes provide orientation for the interpretation of effects in terms of clinical or epidemiological relevance.

### Sensitivity analyses

In the first sensitivity analysis, we restricted the study population to participants with no known malignant diseases at the time of the SHIP examination. Participants in which this information was unknown were also excluded. The interview in which this information was reported by SHIP participants antedates any disclosure of IFs. This analysis was conducted to control for the impact of known malignant conditions on the number of biopsies after SHIP participation.

In the second sensitivity analysis, we examined the coverage of biopsies based on participant place of residence. The University Department of Pathology is the main biopsy provider in the region, covering all inpatient and a portion of outpatient biopsies. Nevertheless, biopsies may be sent to private laboratories in the region, particularly for patients living far away from Greifswald University Medical Center. Similarly, the decision to participate in wb-MRI may have depended on the distance of participants’ residence to the examination center. Both of these potentially limiting factors in our study’s coverage were examined using stratification for distance to the examination center.

## Results

### Participant characteristics

The descriptive characteristics of participants (N = 6753) are summarized in Table 1 stratified by cohort and MRI participation. Participants who underwent MRI were younger compared to non-participants, had a higher education and employment status, and were more frequently in a relationship. The percentage of biopsy reports was similar across all four strata and ranged from 17.2 to 18.3%. Disclosure of abnormal laboratory findings was more frequent in MRI non-participants. In total, 1022 out of 3371 participants (30.3%) who underwent MRI examination (SHIP-2: n = 362, SHIP-TREND-0: n = 660) received disclosure of an incidental MRI finding. Of these 851 (83.3%) were suspected to be tumors.

### Frequency of biopsies

Within ± 2 years to SHIP there were 2271 histological reports belonging to 1200 participants. The reports were dated from 2006 to 2012. Of these, 938 biopsy reports (599 participants) occurred in the 2 years prior to the SHIP examination and 1333 (739 participants) occurred during the 2 years after SHIP. A detailed list of biopsies and outcomes is shown in Table A of the Online Appendix. These numbers correspond to an event rate of biopsies of 6.9 [95% CI 6.5; 7.4] pre-SHIP and 9.9 [9.3; 10.4] post-SHIP (rate per 100 observation years).

For 601 participants (50.0%), the first recorded biopsy was observed after participation in SHIP; i.e., no biopsy had been recorded in the Department of Pathology between 2002 and the date of the SHIP examination. Of the 1333 biopsies found in the 2 years after participation in SHIP, 1041 (78.1%) belonged to these 601 participants.

Factors associated with a higher number of biopsies included higher age, female sex, hospitalization (within 12 months of SHIP examination), history of cancer, and the disclosure of IFs (laboratory and MRI), as well as time (Table 2). No or only minor effects were associated with education, relationship status and employment status. In a model stratified for sex the IRRs were comparable (Online Appendix, Table B). In model 2 only IFs from MRI with tumor relevance (Yes vs. No) were considered. This analysis shows a small increase of the effect size for MRI IFs.

**Table 2:** Predictors for the no. of biopsy reports

Predictors for biopsy reports	Model 1		Model 2	
	IRR	95% CI	IRR	95% CI
Age (per decade)	1.15	[1.08; 1.23]	1.16	[1.09; 1.23]
Sex (male vs. female)	0.73	[0.63; 0.84]	0.73	[0.63; 0.85]
Education (years, reference: 10y)				
< 10y	0.99	[0.81; 1.20]	0.99	[0.82; 1.2]
> 10y	0.99	[0.83; 1.17]	0.99	[0.84; 1.17]
Employed (yes vs. no)	0.92	[0.77; 1.11]	0.93	[0.77; 1.11]
Relationship status (reference: single)				
Married	1.04	[0.80; 1.35]	1.03	[0.79; 1.34]
Divorced	0.94	[0.65; 1.35]	0.92	[0.64; 1.33]
Widowed	1.17	[0.79; 1.75]	1.16	[0.77; 1.73]
Hospitalized in last 12 months (yes vs. no)	3.45	[3.01; 3.96]	3.45	[3.01; 3.96]
Known cancer history (yes vs. no)	2.89	[2.28; 3.67]	2.89	[2.28; 3.67]
Time-varying measures				
Disclosure of lab anomaly (yes vs. no)	1.37	[1.12; 1.67]	1.37	[1.12; 1.66]
Disclosure of MRI IF (yes vs. no)	2.17	[1.76; 2.68]	2.32	[1.85; 2.89]
Time (post-SHIP vs. pre-SHIP)	1.29	[1.07; 1.55]	1.30	[1.08; 1.56]

Results from GEE with a negative binomial distribution shown as incidence-rate-ratios (IRR). In model 2 only IFs from MRI with tumor relevance were used

GEE with a negative binomial distribution calculated in n = 6593 participants due to missing data in covariates

### Biopsy outcomes

Both before and after SHIP examination, the majority of biopsies resulted in the outcome “no malignancy or tumor” (Table 3). The largest absolute increase was found in this category. Biopsies diagnosing a malignancy increased irrespective of the disclosure of IFs in all strata, but rate ratios were highest in case of disclosed MRI findings. Participants with disclosed laboratory or MRI IFs received more biopsies in all outcome categories except benign tumors.

**Table 3:** Outcomes of biopsies stratified for disclosure of incidental findings

Outcome	Combination of IFs	Strata size (participants)	Pre SHIP N biopsies (participants)	Post SHIP N biopsies (participants)	Rate ratio biopsies [CI]	Δ participants (%)
No malignancy or tumor	Lab−|MRI−	2046	127 (109)	138 (119)	1.09 [0.85; 1.38]	10 (0.49)
	Lab+|MRI−	3685	316 (245)	385 (286)	1.22 [1.05; 1.41]	41 (1.11)
	Lab−|MRI+	396	31 (29)	58 (46)	1.87 [1.21; 2.92]	37 (9.34)
	Lab+|MRI+	626	66 (51)	111 (82)	1.68 [1.24; 2.29]	31 (4.95)
Benign tumor	Lab−|MRI−	2046	18 (18)	20 (17)	1.11 [0.58; 2.13]	− 1 (− 0.05)
	Lab+|MRI−	3685	39 (36)	32 (32)	0.82 [0.51; 1.31]	− 4 (− 0.11)
	Lab−|MRI+	396	11 (10)	12 (12)	1.09 [0.47; 2.53]	2 (0.51)
	Lab+|MRI+	626	17 (14)	25 (20)	1.47 [0.79; 2.77]	6 (0.96)
Pre-cancerous lesion (including carcinoma in situ)	Lab−|MRI−	2046	16 (14)	11 (9)	0.69 [0.31; 1.49]	− 5 (− 0.24)
	Lab+|MRI−	3685	33 (30)	50 (43)	1.51 [0.98; 2.37]	13 (0.35)
	Lab−|MRI+	396	2 (1)	7 (7)	3.32 [0.78; 24.59]	6 (1.52)
	Lab+|MRI+	626	10 (8)	13 (11)	1.30 [0.56; 3.06]	3 (0.48)
Malignant process	Lab−|MRI−	2046	16 (16)	27 (23)	1.68 [0.91; 3.20]	7 (0.34)
	Lab+|MRI−	3685	41 (38)	63 (55)	1.53 [1.04; 2.29]	17 (0.46)
	Lab−|MRI+	396	3 (3)	12 (12)	3.85 [1.21; 17.67]	9 (2.27)
	Lab+|MRI+	626	15 (13)	28 (26)	1.86 [1.00; 3.58]	13 (2.08)

Lab−|MRI−, no disclosure of laboratory or MRI IFs; Lab+|MRI−, disclosure of laboratory IFs and no disclosure of MRI IFs; Lab−|MRI+, no disclosure of laboratory IFs and disclosure of MRI IFs; Lab+|MRI+, disclosure of laboratory; MRI IFs Δ, delta or change in the number of participants

### Cumulative rate of selected outcomes

**Fig. 2:**
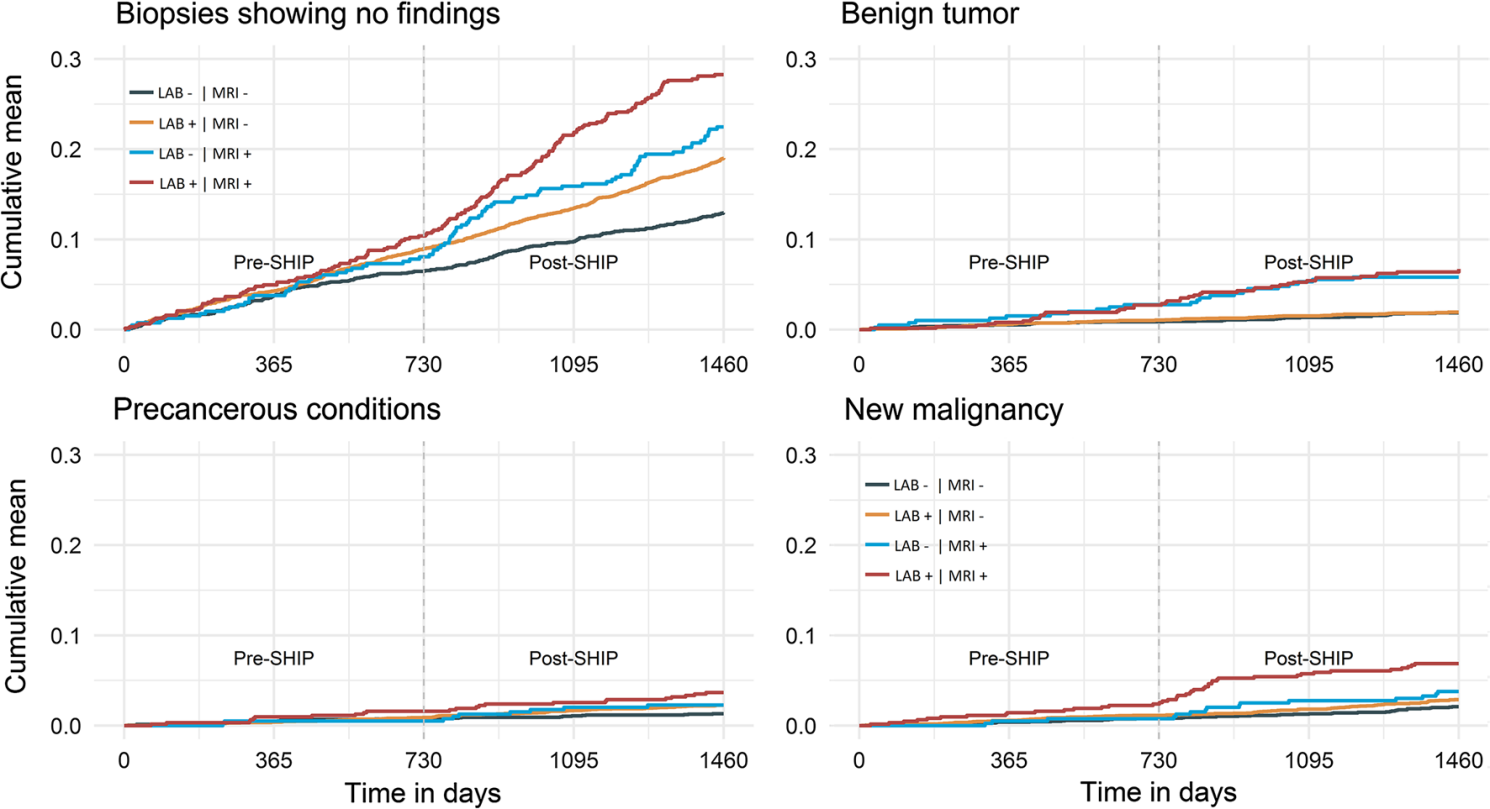
Cumulative biopsy rates. Cumulative rates of biopsies identifying (top left) exclusion of malignancy or benign tumor, (top right) benign tumors, (bottom left) pre-cancerous conditions (including carcinoma in situ), and (bottom right) malignancies

Across all biopsy outcome categories, participants with disclosed MRI findings consistently showed the greatest increase in cumulative biopsy rates post-SHIP. Participants who received a combination of laboratory and MRI IFs were more likely to undergo a biopsy resulting in the diagnosis of a malignancy (Fig. 2).

### Sensitivity analysis

In the first set of sensitivity analyses, we excluded n = 455 participants with known malignant disease and n = 131 participants in which this information was unknown. The impact of IF disclosure was found to be higher than in the results obtained in the unselected study population (Online Appendix, Table C, D and Figure A).

Regarding coverage, MRI participation decreased slightly but inconsistently with increasing distance of participants’ residence to the examination center (55–43%, Online Appendix: Table E) and has therefore not been considered in separate analysis. Whereas biopsy data from the Department of Pathology was available for 22% of participants living near the examination site, only 7.5% of participants living 40–89 km away had available biopsy data (Online Appendix, Table F).

We applied two approaches to analyze the sensitivity of our results to potentially missing biopsy reports. First, we restricted the GEE to participants living close to the examination center. Second, we conducted a weighted GEE, i.e., biopsies of participants living further away were given higher weights. In both approaches, changes in coefficients were marginal and relevant predictors for an increase of biopsies remained the same (Online Appendix, Table G).

## Discussion

We assessed the effects of the disclosure of MRI and laboratory IFs on the frequency and outcomes of biopsies in the 2 years after examination in a large population-based cohort study. The overall number of biopsies increased by 42.7% comparing the 2 years before SHIP examination with the 2 years afterwards. The disclosure of MRI and laboratory IFs contributed strongly to this increase; in particular, MRI IFs with tumor relevance (Table 2, model 2). IF disclosures are therefore likely to reflect an undesired intervention effect in our observational study. New malignancies were most often discovered in a subgroup of participants with laboratory abnormalities and MRI disclosures. However, the absolute number of malignancies detected due to biopsies was small compared to the number of biopsies yielding no malignancy or tumor. Therefore, from a clinical perspective, overtesting and potentially overdiagnosis seem to be negative consequences associated with the disclosure of incidental findings in the setting of a general population cohort. Our findings favor a more restrictive communication policy of incidental study findings.

While the number of biopsies was expected to rise in an aging cohort, this is unlikely to account for the increase we observed. A more plausible explanation is that the disclosed IFs played a causative role in the increase in biopsies. First, the written disclosure of an MRI finding always included a recommendation to consult a doctor. Second, the vast majority of disclosed findings was related to tumors of unknown clinical significance [1] which may trigger biopsies. The overall effect of disclosed MRI IFs on biopsies was larger than that of laboratory abnormalities. This is plausible as the disclosure of laboratory findings did not entail any explicit recommendation to seek further diagnostic action.

The absolute effect of IF disclosure, both for imaging and laboratory findings, was greatest for biopsies showing nonmalignant findings. More than every fourth participant with an MRI and laboratory IF (28.1%) had at least one biopsy, and 62.1% of biopsies revealed no malignancy or tumor. In comparison, few new malignancies were detected via biopsy after SHIP. The low rate of malignancies detected in our large population-based cohort study corresponds to results from smaller studies using wb-MRI. In a study of 666 subjects the incidence of malignant lesions was 1.05%; in 83 subjects this number was 2.40% [34, 35]. An umbrella review found that malignancy rates of IF from clinical imaging range from less than 5–42% [2]. However, most of the studies in this review included asymptomatic oncology patients, explaining the higher rate of malignancies compared to our population-based study.

The effects of the disclosure of MRI and laboratory IFs on biopsies revealing benign and precancerous conditions was less consistent. This may be due to underpowered analyses given the low number of participants and events (biopsies) in the subgroups as defined by different combinations of IF types. However, increases in the detection of benign and precancerous conditions may also represent an indirect effect of IF disclosure, namely the triggering of so-called cascades of care [36]. Benign and precancerous conditions are generally not expected to cause laboratory anomalies, and small precancerous lesions are not expected to be visible on MRI. However, the disclosure of any type of IF has been shown to initiate a range of diagnostic tests, often resulting in additional diagnoses of varying clinical significance [17, 36].

From a clinical perspective, some participants may have benefited from disclosed IFs through the early detection of new malignancies, most of which were diagnosed within 6 months after the SHIP examination. However, the clinical benefit of the earlier detection of malignancies is uncertain, as early diagnosis and intervention does not always equate to a better outcome [10]. We cannot determine from our data which of the disclosed IFs led to therapeutic benefits. Furthermore, the high rate of biopsies resulting in no malignant findings raises the risk for harm resulting from cascades of care, in which patients are exposed to the additional risks and complications of unnecessary tests and interventions [17, 36]. Overdetected conditions have the potential to increase diverse types of costs to the participant and the health care system [7]. These include, among others, psychosocial and financial burdens [8, 9, 37]. In addition, biopsies may be part of a larger cascade of care with preceding diagnostic steps such as further imaging or laboratory tests triggered by IFs [36]. As a result, we likely underestimate the burden of overtesting that resulted from IF disclosure. Clinicians and patients faced with IFs often feel compelled to pursue cascades of care to rule out serious disease out of a need for certainty [38]. Our results serve as a reminder to clinicians to critically assess the need and consequences of diagnostic tests prior to ordering them.

From a research perspective, the disclosure of IFs introduced bias into health service utilization and other outcomes [8, 37] in our population-based cohort study. This loss in validity needs to be weighed against the well-being and health of our study participants. Our results suggest that the benefit of the IF disclosure is limited at best. Moreover, many participants reported distress due to IF disclosure [9]. In the context of the limited benefits associated with IF disclosure, we recommend a restrictive disclosure policy for research studies to minimize the costs and consequences for participants and the health care system.

### Strengths and limitations

To the best of our knowledge, this is the first study that has used routine biopsies as an outcome measure of IF disclosure. By combining cross-sectional research data with longitudinal routine clinical data on invasive procedures following disclosure of MRI IFs, our study contributes to a better understanding of the consequences of IF disclosure in research and clinical settings. All findings in this study are considered incidental as they were obtained outside a clinical context of routine care. Nonetheless, not all findings were new to the participants because of clinical diagnostics outside the SHIP study. Yet, only a minority of 13.3% in a surveyed SHIP-subsample reported already having had full knowledge of the communicated findings [9].

A limitation of this study is the missing direct link between an IF and the observed biopsies. Organ-specific IFs should result in specific biopsies and this association has not been examined in detail in this study (Online Appendix, Figure C). Rather, our approach examines a global association between IFs and changes in the number of biopsies. Furthermore, the presence of known malignant disease in some participants (overall: n = 586, MRI participants: n = 238) implies that some IFs cannot be considered truly incidental as the biopsies conducted after SHIP may have been a consequence of this disease. Nevertheless, the impact of IF disclosure on the number of biopsies conducted remained robust in sensitivity analyses including only participants with no previously known malignant diseases.

We are aware that certain biopsies (e.g., Pap-smears) were performed in an ambulatory setting and are missing from our data. Biopsies for patients living far away from the study site may have been more often conducted in pathology laboratories other than the Department of Pathology. However, we have no reason to assume a systematic difference between wb-MRI participants and non-participants in this regard. Our results remained robust in a sensitivity analysis including only participants living near our site.

The cumulative availability of biopsy reports from 2002 onward is consistent (Online Appendix, Figure B), but a small number of biopsy reports may have been lost in the linkage process. We have no reason to assume a systematic linkage error affecting any one subgroup. We assume that the small proportion of reports (16.6%) that did not undergo a double reading do not affect our results because misclassification in the cross-validated reports was less than 5%.

## Conclusion

The disclosure of MRI and laboratory findings in a population-based cohort study is fraught with problems for both participants and the integrity of observational research. The disclosure of IFs represents an intervention that introduced bias into the natural course of health service utilization by exerting a lasting influence on biopsy rates. Researchers and clinicians should be aware that the increase in biopsy frequency after disclosure of MRI and laboratory IFs is substantial, but the rate of malignancies diagnosed is low. Our data thus supports a more restrictive disclosure policy for research MRI findings.

## Electronic supplementary material

Below is the link to the electronic supplementary material.

Supplementary material 1 (DOCX 432 kb)

## Data Availability

Data of the SHIP studies are available and can be applied for under https://www.fvcm.med.uni-greifswald.de/dd_service/data_use_intro.php. The histology data utilized in this study are not publicly available due to privacy restrictions to protect personal data of research participants.
