# Supplementary material for: The effects of incidental findings from whole-body MRI on the frequency of biopsies and detected malignancies or benign conditions in a general population cohort study
Source: Eur J Epidemiol. 2020 Aug 29;35(10):925–35. doi: 10.1007/s10654-020-00679-4 (PMC7524843; doi:10.1007/s10654-020-00679-4)
Supplement: Supplementary file 1 — Supplementary material 1 (DOCX 432 kb) [file 10654_2020_679_MOESM1_ESM.docx]

Supplementary Tables and Figures

**Table A:** Detailed outcomes of biopsies stratified for the disclosure of incidental findings. 2

**Table B:** GEE results stratified for participants’ sex shown as incidence-rate-ratios (IRR). 3

**Table C:** GEE results stratified for the subsample of participants without known malignancies (IRR). 3

**Table D:** Outcomes of biopsies stratified for disclosure of incidental findings in participants without known malignancies. 4

**Table E:** MRI participation stratified by distance of participants’ residence to SHIP center. 4

**Table F:** Availability of biopsies stratified by distance of participants’ residence to SHIP center. 4

**Table G:** Results of sensitivity analyses for the coverage of biopsy reports. 5

**Figure A:** Cumulative biopsy rates in participants without known malignancies. 6

**Figure B:** Availability of biopsies after linkage to SHIP data. 7

**Figure C:** The no. of biopsies stratified for different organ systems. 8

# **Table A:** Detailed outcomes of biopsies stratified for the disclosure of incidental findings.

| **Outcomes** | **Strata** | **N  Participants** | **N Biopsies  (Participants) pre SHIP** | **N Biopsies  (Participants) post SHIP** | **Biopsy RR [CI]** |
| --- | --- | --- | --- | --- | --- |
| No malignancy or tumor | Lab - \| MRI - | 2046 | 127 (109) | 138 (119) | 1.09 [0.85; 1.38] |
|  | Lab + \| MRI - | 3685 | 316 (245) | 385 (286) | 1.22 [1.05; 1.41] |
|  | Lab - \| MRI + | 396 | 31 (29) | 58 (46) | 1.87 [1.21; 2.92] |
|  | Lab + \| MRI + | 626 | 66 (51) | 111 (82) | 1.68 [1.24; 2.29] |
| Benign tumor | Lab - \| MRI - | 2046 | 18 (18) | 20 (17) | 1.11 [0.58; 2.13] |
|  | Lab + \| MRI - | 3685 | 39 (36) | 32 (32) | 0.82 [0.51; 1.31] |
|  | Lab - \| MRI + | 396 | 11 (10) | 12 (12) | 1.09 [0.47; 2.53] |
|  | Lab + \| MRI + | 626 | 17 (14) | 25 (20) | 1.47 [0.79; 2.77] |
| Pre-cancerous lesion  (including carcinoma in situ) | Lab - \| MRI - | 2046 | 16 (14) | 11 (9) | 0.69 [0.31; 1.49] |
|  | Lab + \| MRI - | 3685 | 33 (30) | 50 (43) | 1.51 [0.98; 2.37] |
|  | Lab - \| MRI + | 396 | 2 (1) | 7 (7) | 3.32 [0.78; 24.59] |
|  | Lab + \| MRI + | 626 | 10 (8) | 13 (11) | 1.3 [0.56; 3.06] |
| 1st malignancy | Lab - \| MRI - | 2046 | 15 (15) | 21 (21) | 1.4 [0.72; 2.77] |
|  | Lab + \| MRI - | 3685 | 33 (33) | 44 (44) | 1.33 [0.85; 2.11] |
|  | Lab - \| MRI + | 396 | 2 (2) | 11 (11) | 5.18 [1.37; 36.5] |
|  | Lab + \| MRI + | 626 | 11 (11) | 21 (21) | 1.9 [0.93; 4.11] |
| 2nd malignancy | Lab - \| MRI - | 2046 | 1 (1) | 2 (2) | 1.88 [0.15; 58.99] |
|  | Lab + \| MRI - | 3685 | 3 (3) | 10 (10) | 3.22 [0.97; 15.05] |
|  | Lab - \| MRI + | 396 | 0 (0) | 0 (0) | 0 [0; Inf] |
|  | Lab + \| MRI + | 626 | 2 (2) | 4 (4) | 1.93 [0.35; 15.61] |
| 3rd malignancy | Lab - \| MRI - | 2046 | 0 (0) | 1 (1) | NA |
|  | Lab + \| MRI - | 3685 | 1 (1) | 2 (2) | 1.88 [0.15; 58.99] |
|  | Lab - \| MRI + | 396 | 1 (1) | 0 (0) | 0 [0; 19] |
|  | Lab + \| MRI + | 626 | 0 (0) | 1 (1) | NA |
| 4th malignancy | Lab - \| MRI - | 2046 | 0 (0) | 1 (1) | NA |
|  | Lab + \| MRI - | 3685 | 1 (1) | 0 (0) | 0 [0; 19] |
|  | Lab - \| MRI + | 396 | 0 (0) | 0 (0) | 0 [0; Inf] |
|  | Lab + \| MRI + | 626 | 0 (0) | 0 (0) | 0 [0; Inf] |
| 5th malignancy | Lab - \| MRI - | 2046 | 0 (0) | 0 (0) | 0 [0; Inf] |
|  | Lab + \| MRI - | 3685 | 1 (1) | 0 (0) | 0 [0; 19] |
|  | Lab - \| MRI + | 396 | 0 (0) | 0 (0) | 0 [0; Inf] |
|  | Lab + \| MRI + | 626 | 0 (0) | 0 (0) | 0 [0; Inf] |
| Metastatis | Lab - \| MRI - | 2046 | 0 (0) | 2 (2) | NA |
|  | Lab + \| MRI - | 3685 | 2 (1) | 7 (7) | 3.32 [0.78; 24.59] |
|  | Lab - \| MRI + | 396 | 0 (0) | 1 (1) | NA |
|  | Lab + \| MRI + | 626 | 2 (1) | 2 (2) | 1 [0.1; 9.61] |
| Consecutive report | Lab - \| MRI - | 2046 | 24 (19) | 32 (20) | 1.33 [0.79; 2.29] |
|  | Lab + \| MRI - | 3685 | 42 (31) | 98 (54) | 2.33 [1.63; 3.38] |
|  | Lab - \| MRI + | 396 | 3 (3) | 20 (14) | 6.38 [2.17; 28.2] |
|  | Lab + \| MRI + | 626 | 11 (7) | 39 (24) | 3.51 [1.86; 7.23] |
| Follow-up of known  malignant or  suspicious process | Lab - \| MRI - | 2046 | 33 (17) | 31 (21) | 0.94 [0.57; 1.54] |
|  | Lab + \| MRI - | 3685 | 44 (29) | 80 (41) | 1.82 [1.26; 2.64] |
|  | Lab - \| MRI + | 396 | 3 (2) | 11 (6) | 3.53 [1.09; 16.36] |
|  | Lab + \| MRI + | 626 | 16 (11) | 29 (18) | 1.8 [0.99; 3.41] |
| **Total** |  | **6753** | **938 (599)** | **1333 (739)** | **1.42 [1.31; 1.55]** |

Lab - | MRI - = no disclosure of laboratory or MRI IFs, Lab + | MRI - = disclosure of laboratory IFs and no disclosure of MRI IFs, Lab - | MRI + = no disclosure of laboratory IFs and disclosure of MRI IFs, Lab + | MRI + = disclosure of laboratory and MRI IFs.

# **Table B:** GEE results stratified for participants’ sex shown as incidence-rate-ratios (IRR).

|  | FEMALES | | | MALES | | |
| --- | --- | --- | --- | --- | --- | --- |
|  | IRR | LCL | UCL | IRR | LCL | UCL |
| (Intercept) | 0.06 | 0.04 | 0.10 | 0.02 | 0.01 | 0.03 |
| Age (per decade) | 1.07 | 1.00 | 1.15 | 1.29 | 1.14 | 1.46 |
| Education (years, reference: 10y) |  |  |  |  |  |  |
| <10y | 0.84 | 0.63 | 1.10 | 1.20 | 0.91 | 1.58 |
| >10y | 0.95 | 0.75 | 1.21 | 1.03 | 0.80 | 1.33 |
| Relationship status (reference: single) |  |  |  |  |  |  |
| Married | 1.09 | 0.74 | 1.60 | 0.90 | 0.61 | 1.31 |
| Divorced | 1.03 | 0.63 | 1.68 | 0.79 | 0.45 | 1.38 |
| Widowed | 1.30 | 0.78 | 2.16 | 1.23 | 0.61 | 2.46 |
| Work status (yes vs. no) | 0.97 | 0.77 | 1.23 | 0.90 | 0.68 | 1.20 |
| Known cancer history (yes vs. no) | 2.90 | 2.13 | 3.95 | 2.80 | 1.87 | 4.21 |
| Hospitalization in last 12 month (yes vs. no) | 3.29 | 2.74 | 3.95 | 3.69 | 2.99 | 4.56 |
| Disclosure of laboratory IFs (yes vs. no) | 1.50 | 1.14 | 1.96 | 1.25 | 0.94 | 1.66 |
| Disclosure of MRI IFs (yes vs. no) | 1.87 | 1.41 | 2.48 | 2.46 | 1.78 | 3.39 |
| Time (post SHIP vs. pre SHIP) | 1.21 | 0.94 | 1.56 | 1.37 | 1.05 | 1.79 |

# **Table C:** GEE results stratified for the subsample of participants without known malignancies (IRR).

|  | Model 1 | | Model 2 | |
| --- | --- | --- | --- | --- |
| Predictors for biopsy reports | IRR | 95% CI | IRR | 95% CI |
| Age (per decade) | 1.2 | [1.13; 1.28] | 1.21 | [1.13; 1.29] |
| Sex (male vs. female) | 0.70 | [0.61; 0.82] | 0.70 | [0.61; 0.82] |
| Education (years, reference: 10y) |  |  |  |  |
| <10y | 1.02 | [0.82; 1.25] | 1.02 | [0.83; 1.26] |
| >10y | 1.03 | [0.86; 1.24] | 1.04 | [0.87; 1.24] |
| Employed (yes vs. no) | 0.93 | [0.77; 1.12] | 0.94 | [0.77; 1.13] |
| Relationship status (reference: single) |  |  |  |  |
| Married | 1.01 | [0.77; 1.34] | 1.00 | [0.76; 1.32] |
| Divorced | 0.93 | [0.63; 1.38] | 0.91 | [0.61; 1.35] |
| Widowed | 1.05 | [0.7; 1.59] | 1.04 | [0.69; 1.58] |
| Hospitalized in last 12 months (yes vs. no) | 3.71 | [3.2; 4.29] | 3.70 | [3.2; 4.29] |
| Known cancer history (yes vs. no) | NA | NA | NA | NA |
| *Time-varying measures* |  |  |  |  |
| Disclosure of lab anomaly (yes vs. no) | 1.38 | [1.12; 1.69] | 1.38 | [1.12; 1.7] |
| Disclosure of MRI IF (yes vs. no) | 2.23 | [1.78; 2.79] | 2.37 | [1.87; 3] |
| Time (post-SHIP vs. pre-SHIP) | 1.44 | [1.18; 1.75] | 1.45 | [1.19; 1.76] |

GEE with a negative binomial distribution calculated in n=6,167 participants due to missing data in covariates and due to the exclusion of participants with known malignant diseases (n=586).

# **Table D:** Outcomes of biopsies stratified for disclosure of incidental findings in participants without known malignancies.

| **Outcome** | **Combination**  **of IFs** | **Strata size (Participants)** | | **Pre SHIP N Biopsies  (Participants)** | | **Post SHIP N Biopsies  (Participants)** | | | **Rate ratio  biopsies [CI]** | |
| --- | --- | --- | --- | --- | --- | --- | --- | --- | --- | --- |
|  |  | **unselected** | **selected** | |  | |  |  | |  |
| No malignancy or tumor | Lab - \| MRI - | 2046 | **1883** | | 118 (100) | | 127 (108) | 1.08 (0.84; 1.38) | |  |
|  | Lab + \| MRI - | 3685 | **3353** | | 264 (212) | | 338 (248) | 1.28 (1.09; 1.50) | |  |
|  | Lab - \| MRI + | 396 | **372** | | 31 (29) | | 51 (41) | 1.64 (1.06; 2.60) | |  |
|  | Lab + \| MRI + | 626 | **559** | | 53 (40) | | 101 (74) | 1.90 (1.37; 2.67) | |  |
| Benign tumor | Lab - \| MRI - | 2046 | **1883** | | 16 (16) | | 14 (11) | 0.88 (0.42; 1.81) | |  |
|  | Lab + \| MRI - | 3685 | **3353** | | 35 (33) | | 32 (32) | 0.91 (0.56; 1.48) | |  |
|  | Lab - \| MRI + | 396 | **372** | | 11 (10) | | 10 (10) | 0.91 (0.38; 2.18) | |  |
|  | Lab + \| MRI + | 626 | **559** | | 12 (10) | | 23 (19) | 1.90 (0.96; 3.98) | |  |
| Pre-cancerous lesion  (including carcinoma in situ) | Lab - \| MRI - | 2046 | **1883** | | 13 (11) | | 9 (8) | 0.70 (0.28; 1.63) | |  |
|  | Lab + \| MRI - | 3685 | **3353** | | 22 (20) | | 42 (36) | 1.90 (1.15; 3.25) | |  |
|  | Lab - \| MRI + | 396 | **372** | | 2 (1) | | 6 (6) | 2.86 (0.63; 21.59) | |  |
|  | Lab + \| MRI + | 626 | **559** | | 4 (4) | | 12 (10) | 2.92 (1.00; 10.77) | |  |
| Malignant process | Lab - \| MRI - | 2046 | **1883** | | 3 (3) | | 26 (22) | 8.28 (2.90; 36.04) | |  |
|  | Lab + \| MRI - | 3685 | **3353** | | 9 (9) | | 51 (43) | 5.58 (2.88; 12.21) | |  |
|  | Lab - \| MRI + | 396 | **372** | | 1 (1) | | 11 (11) | 9.74 (1.88; 240.04) | |  |
|  | Lab + \| MRI + | 626 | **559** | | 2 (2) | | 21 (19) | 9.82 (2.87; 66.48) | |  |

**Strata size: selected = participants with known malignant diseases (n=586) were excluded**. Lab - | MRI - = no disclosure of laboratory or MRI IFs, Lab + | MRI - = disclosure of laboratory IFs and no disclosure of MRI IFs, Lab - | MRI + = no disclosure of laboratory IFs and disclosure of MRI IFs, Lab + | MRI + = disclosure of laboratory and MRI IFs Δ = delta or change in the number of participants

# **Table E:** MRI participation stratified by distance of participants’ residence to SHIP center.

| Distance to SHIP | No MRI (N) | MRI (N) | Percentage of participation (%) |
| --- | --- | --- | --- |
| <10km | 816 | 1005 | 55.19 |
| 10-19km | 308 | 378 | 55.10 |
| 20-29km | 379 | 282 | 42.66 |
| 30-39km | 1289 | 1186 | 47.92 |
| 40-49km | 284 | 216 | 43.20 |
| 50-89km | 141 | 111 | 44.05 |
| above 89km | 165 | 193 | 53.91 |

# **Table F:** Availability of biopsies stratified by distance of participants’ residence to SHIP center.

| **Distance to SHIP** | **Histological data: No** | **Histological data: Yes** | **Percentage (%)** | **Weight** | **N** | **Prob** |
| --- | --- | --- | --- | --- | --- | --- |
| <10km | 1421 | 400 | 21.97 | 1.00000000 | 1821 | 0.26965793 |
| 10-19km | 549 | 137 | 19.97 | 1.10015023 | 686 | 0.10158448 |
| 20-29km | 519 | 142 | 21.48 | 1.02281192 | 661 | 0.09788242 |
| 30-39km | 2096 | 379 | 15.31 | 1.43500980 | 2475 | 0.36650378 |
| 40-49km | 408 | 92 | 18.4 | 1.19402174 | 500 | 0.07404117 |
| 50-89km | 229 | 23 | 9.13 | 2.40635268 | 252 | 0.03731675 |
| above 89km | 331 | 27 | 7.54 | 2.91379310 | 358 | 0.05301348 |

# **Table G:** Results of sensitivity analyses for the coverage of biopsy reports.

|  | Weighted GEE | | | GEE restricted to participants  of closest region | | |
| --- | --- | --- | --- | --- | --- | --- |
|  | IRR | LCL | UCL | IRR | LCL | UCL |
| Intercept | 0.06 | 0.04 | 0.09 | 0.05 | 0.02 | 0.10 |
| Age (per decade) | 1.14 | 1.07 | 1.22 | 1.18 | 1.05 | 1.32 |
| Sex (males vs. females) | 0.71 | 0.61 | 0.82 | 0.78 | 0.60 | 1.00 |
| Education (years, reference: 10y) |  |  |  |  |  |  |
| <10y | 1.04 | 0.84 | 1.28 | 0.80 | 0.55 | 1.16 |
| >10y | 0.96 | 0.81 | 1.15 | 1.11 | 0.83 | 1.47 |
| Relationship status (reference: single) |  |  |  |  |  |  |
| Married | 1.00 | 0.75 | 1.33 | 1.08 | 0.68 | 1.71 |
| Divorced | 0.86 | 0.59 | 1.25 | 0.96 | 0.52 | 1.74 |
| Widowed | 1.10 | 0.72 | 1.67 | 1.08 | 0.53 | 2.21 |
| Work status (yes vs. no) | 0.89 | 0.73 | 1.08 | 0.90 | 0.64 | 1.26 |
| Hospitalization in last 12 month (yes vs. no) | 3.36 | 2.92 | 3.86 | 3.25 | 2.56 | 4.13 |
| Known cancer history (yes vs. no) | 2.82 | 2.22 | 3.59 | 3.06 | 2.16 | 4.34 |
| Disclosure of laboratory IFs (yes vs. no) | 1.30 | 1.06 | 1.60 | 1.53 | 1.07 | 2.20 |
| Disclosure of MRI IFs (yes vs. no) | 1.94 | 1.57 | 2.41 | 2.32 | 1.62 | 3.31 |
| Time (post SHIP vs. pre SHIP) | 1.35 | 1.12 | 1.64 | 1.17 | 0.84 | 1.64 |

# **Figure A:** Cumulative biopsy rates in participants without known malignancies.


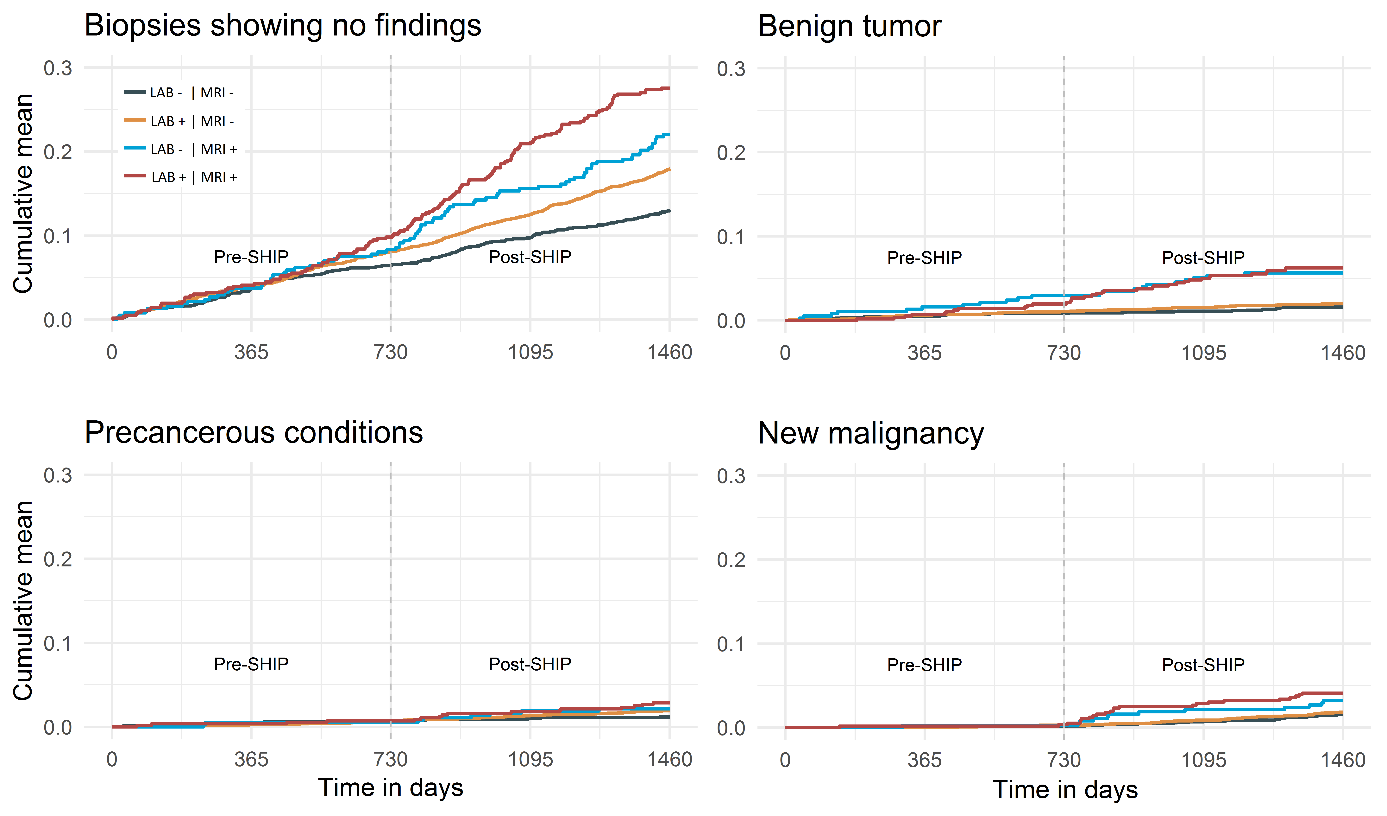


**Participants with known malignant diseases (n=586) were excluded.** Cumulative biopsies after exclusion of participants with known malignant diseases (n=586). Cumulative rates of biopsies identifying (top left) no malignancy or tumor, (top right) benign tumors, (bottom left) pre-cancerous conditions (including carcinoma in situ), and (bottom right) malignancies

# **Figure B:** Availability of biopsies after linkage to SHIP data.

These graphs show the global availability of biopsies for all SHIP-participants. The cumulative growth in panel A is consistent. From overall N=8,576 findings were n=5,999 observed until 2014, the end of the analysis period. From these, n=3,045 were used in the analyses. After exclusion of autopsies and corrupted reports: n=740 were antedating the analysis period, n=938 were found within 2y prior SHIP, and n=1333 within 2y after SHIP.

Since examination in SHIP range over a period of 4-5 years biopsy reports are distributed accordingly (Panel B).

**
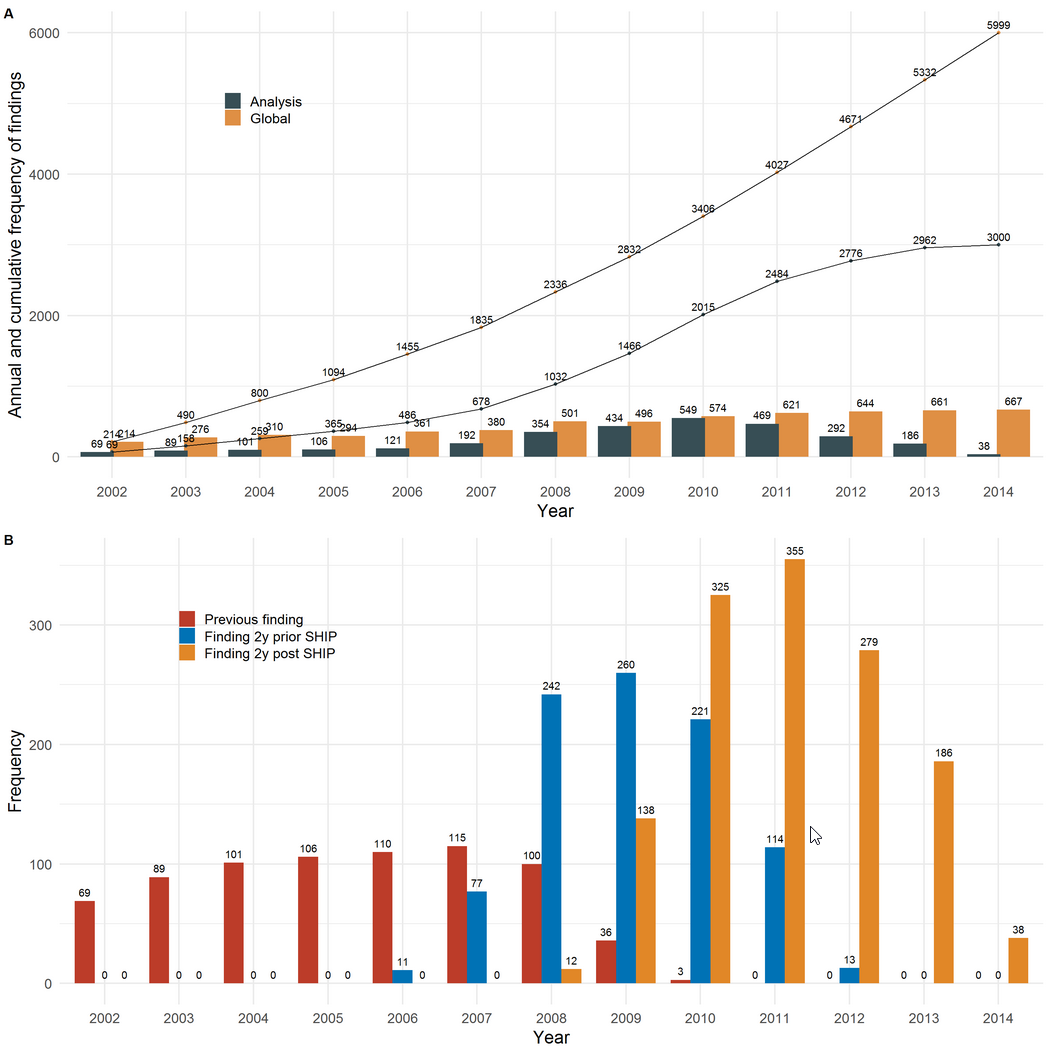
**

# **Figure C:** The no. of biopsies stratified for different organ systems.

*
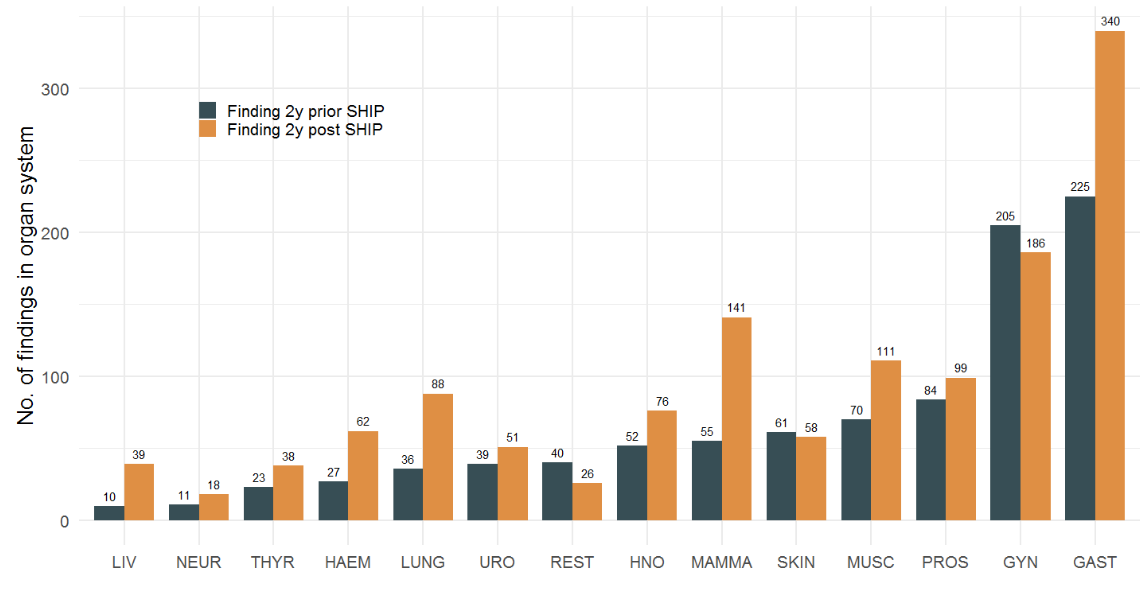
*

Figure 1: Liv = liver and pancreas, NEUR = neurological, THYR = Thyroid, HAEM = hematological, LUNG = pulmonary, URO = urological, REST = miscellaneous, HNO = ear-nose-throat, MAMMA = breast, SKIN = skin, MUSC = musculoskeletal, PROS = prostate, GYN = gynecological, GAST = gastrointestinal
